# Clear cell variant mucoepidermoid carcinoma of salivary gland in a grey wolf (*Canis lupus*)

**DOI:** 10.1002/vms3.909

**Published:** 2022-08-31

**Authors:** Myeon‐Sik Yang, Hyejin Yun, Ki Chang Lee, Chae Woong Lim, Bumseok Kim

**Affiliations:** ^1^ Laboratory of Veterinary Pathology College of Veterinary Medicine Jeonbuk National University Iksan‐si Jeollabuk‐do Republic of Korea; ^2^ Laboratory of Veterinary Radiology College of Veterinary Medicine Jeonbuk National University Iksan‐si Jeollabuk‐do Republic of Korea

**Keywords:** grey wolf, mucoepidermoid carcinoma, zoo animal

## Abstract

A 14‐year‐old male grey wolf (*Canis lupus*) with a history of severe facial swelling was submitted for necropsy. Clinical and radiological examination demonstrated an expansile neoplastic mass in the nasal and frontal sinuses. On necropsy, an amorphous neoplastic mass and extensive necrosis were observed in the nasal turbinate. Microscopic examination revealed a tumour principally composed of obvious clear tumour cells characterised by small hyperchromatic nuclei and abundant clear cytoplasm. These clear cells were positive for mucin with PAS, PAS‐D reaction, and alcian blue (pH 2.5) staining, but negative for PTAH staining. Immunohistochemically, some of tumour cells were strongly positive for mesenchymal cells (vimentin), whereas they were negative for myoepithelial antigen (alpha‐SMA) and cytokeratin. Based on the histopathological and immunohistochemical features, the present case was diagnosed as high‐grade clear cell variant mucoepidermoid carcinoma (MEC). This is the first description of clear cell variant MEC in a wolf.

## Graphical abstract

Mucoepidermoid carcinoma in gray wolf (*Canis lupus*). Extensive osteoblastic and osteolytic changes of the frontal bone and maxillary sinus were observed in radiography. Variously‐sized and ‐shaped clear cells had small hyperchromatic nuclei and abundant clear cytoplasm in histopathological examination.

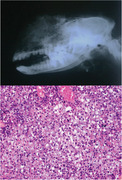

1

Salivary glands are exocrine glands located in the oral cavity of vertebrates. The glands, as the name implies, produce and secrete saliva containing digesting enzymes as well as antibodies. Salivary glands are divided into major and minor salivary glands and are widely distributed over the face (Dyce et al., 2009).

According to the current classification by the World Health Organization (WHO), salivary gland tumours are classified into 10 and 24 specific benign and malignant epithelial tumours in human, respectively (Nagao et al., 2012). However, in veterinary medicine, detailed classification has less importance. For treatment and prognosis evaluation, the focus is on diagnosing malignant and benign (Meuten, 2017). Malignant salivary gland tumours in domestic animals occur more frequently compared to benign tumours, and the most common types are adenocarcinomas and acinic cell tumours (Carberry et al., 1988). These salivary gland tumours are rare in dogs and cats with a reported incidence of only 0.17% of all canine and feline tumours (Carberry et al., 1988; Dorso et al., 2008). In addition to dogs and cats, primary salivary gland tumours have been described in horses, cows, sheep, goats, baboons, guinea pigs, rats and foxes (Carberry et al., 1988).

Mucoepidermoid carcinoma (MEC) is the most common type of malignant epithelial salivary gland tumour (Terada et al., 2004; Varma et al., 2012). In veterinary field, the MEC has been reported in lion (Dorso et al., 2008), Amazon parrot (Nau et al., 2017), Wistar rat (Nolte et al., 1995), goat (Turk et al., 1984), dog and cat (Hammer et al., 2001). Microscopically, MEC is composed of three cell types in varying proportions: mucus‐producing cells, epidermoid (squamoid) cells and undifferentiated intermediate cells (Ettl et al., 2012; Schwarz et al., 2011). In addition to these essential cell types, clear cells are represented in varying proportions in MEC, and some cases have been reported that clear cells infrequently predominate over other cell types (Yoon et al., 2005). Such cases are called clear cell variant MEC (Ellis, 1998). Here we report a rare case of MEC composed of abundant clear cells in a grey wolf (*Canis lupus*).

This case and procedures were reviewed and approved by the Animal Ethics Committee of Jeonbuk National University.

A 14‐year‐old male grey wolf died at the Jeonju Zoo and was referred to the Jeonbuk Veterinary Diagnostic Center for necropsy. The wolf had a five‐month history of severe facial swelling, epistaxis and purulent nasal discharge. The veterinarian of the zoo first suspected an infection of the respiratory system. After that, antibiotics and analgesics were prescribed, and the prognosis was observed.

Grossly, the wolf was emaciated, and the normal physiognomy of the craniofacial area was distorted by severe facial swelling. There were also extensive osteoblastic and osteolytic changes of the frontal bone and maxillary sinus on lateral position radiography. On dorsal ventral radiography, the vomer bone was completely obliterated, the turbinate bone and cribriform cartilage detail were lost, and there were general areas of increased soft tissue opacity in the nasal cavity (Figure 1). Clinical and radiological examination demonstrated an infiltrating neoplastic mass in the nasal and frontal sinuses, with involvement of the periodontal structures.

**FIGURE 1:**
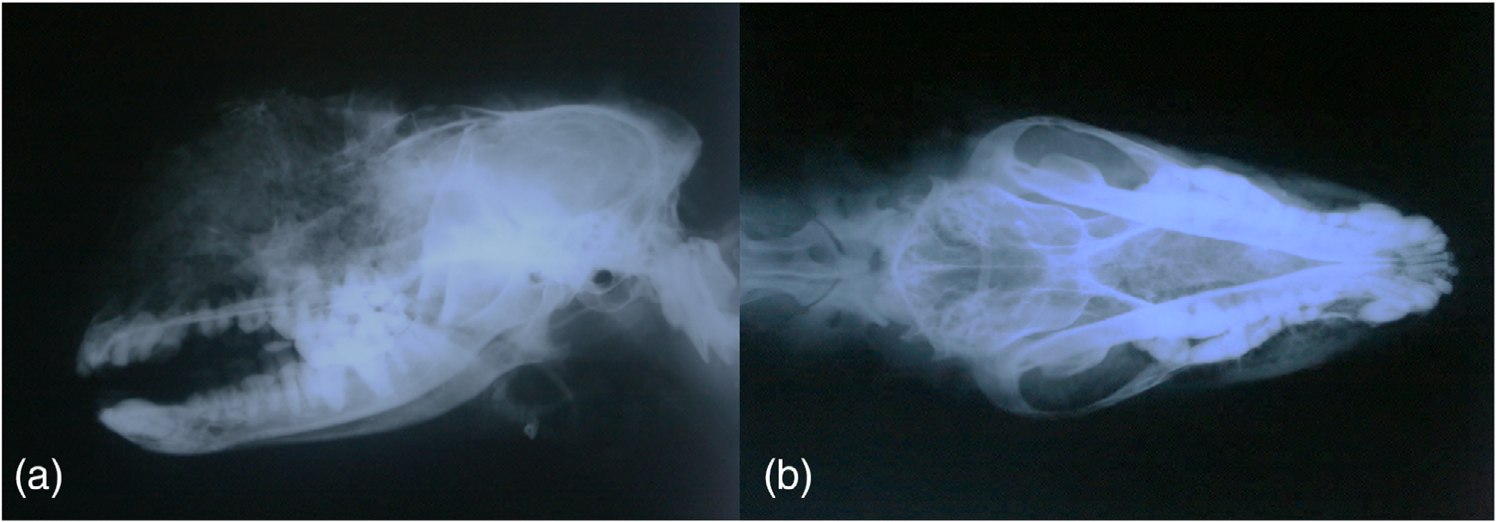
Radiological examination of mucoepidermoid carcinoma, skull, grey wolf (*Canis lupus*). (a) Lateral radiography of the skull. Extensive osteoblastic and osteolytic changes of the frontal bones and maxillary sinuses were observed. (b) Dorsal ventral radiography of the skull. The vomer bone detail had disappeared completely.

At necropsy, necrotic purulent discharge was seen in the maxillary gingivae. After removing the facial skin, a neoplastic mass and extensive necrosis in the nasal turbinate were observed. The amorphous tumour caused destruction of the cribriform plate with infiltration into the cranial vault. No other abnormalities or metastases were detected in other organs. Tissue samples from the mass were collected and fixed in 10% neutral phosphate‐buffered formalin. Fixed tissues were processed, embedded in paraffin, sectioned at 5 μm thickness, and stained with haematoxylin and eosin (H&E), periodic acid‐Schiff (PAS, 395B‐1KT, Sigma‐Aldrich, MO, USA), PAS with diastase (PAS‐D, PAD‐1‐IFU, Scytek, UT, USA), alcian blue (pH 2.5, AFR‐1‐IFU, Scytek, UT, USA) and phosphotungstic acid‐haematoxylin (PTAH, ab150683, Abcam, Cambridge, UK). In addition, immunohistochemical staining with antibodies against vimentin (MA5‐11883, Invitrogen, MA, USA), cytokeratins (CKs, MNF‐116, Dako, CA, USA), and alpha smooth muscle actin (alpha‐SMA, MA1‐06110, Invitrogen, MA, USA) were performed for differential diagnosis. Immunohistochemistry was performed with standard protocol. Antigens were retrieved and heated in buffers at pH 6.0. HRP‐conjugated secondary antibody was applied (MP‐7500, Vector, CA, USA), and 3,3′‐diaminobenzidine (DAB, SK‐4105, Vector, CA, USA) was used as a chromogen. All antibodies were diluted in antibody diluent in ratio with manufacturer's instructions (E09‐300, GBI Labs, WA, USA).

Microscopic examination revealed that the tumour was principally composed of obvious clear tumour cells. Histologically, these variously sized and shaped clear cells had small hyperchromatic nuclei and abundant clear cytoplasm. Some of the tumour cells were round to oval in shape and contained small, dark‐staining nuclei and eosinophilic cytoplasm (Figure 2a). They were regarded as intermediate cells, and these cells appeared to differentiate toward clear cells. A few epidermoid and intermediate cell components were irregularly lined with sheets of clear cells (Figure 2b). In addition, multiple areas of necrosis and cellular atypia were observed; however, abnormal mitotic figures were rarely seen in tumour cells. In the present case, clear cells constituted the major component of this tumour (approximately 90%). These clear cells were positive for mucin with PAS, PAS‐D reaction and alcian blue (pH 2.5) staining (Figure 2c and d). Mucinous material was found within the cytoplasm and extravasating into the stroma. PTAH, which confirms oncocytic mitochondrial activity (Ellis, 1998), was not expressed in the tumour area (Figure 2e). Immunohistochemically, some of tumour cells were strongly positive for mesenchymal cells (vimentin) (Figure 2f), whereas they were negative for myoepithelial antigens such as alpha‐SMA and CKs (data not shown). MEC tumours are usually negative for alpha‐SMA and positive for vimentin (Zhu et al., 2015). Vimentin expression also distinguishes MEC from squamous cell carcinoma (SCC) and adenocarcinoma. These histologic studies were helpful in differentiating MEC from other types of salivary gland tumours with clear cell morphology.

**FIGURE 2:**
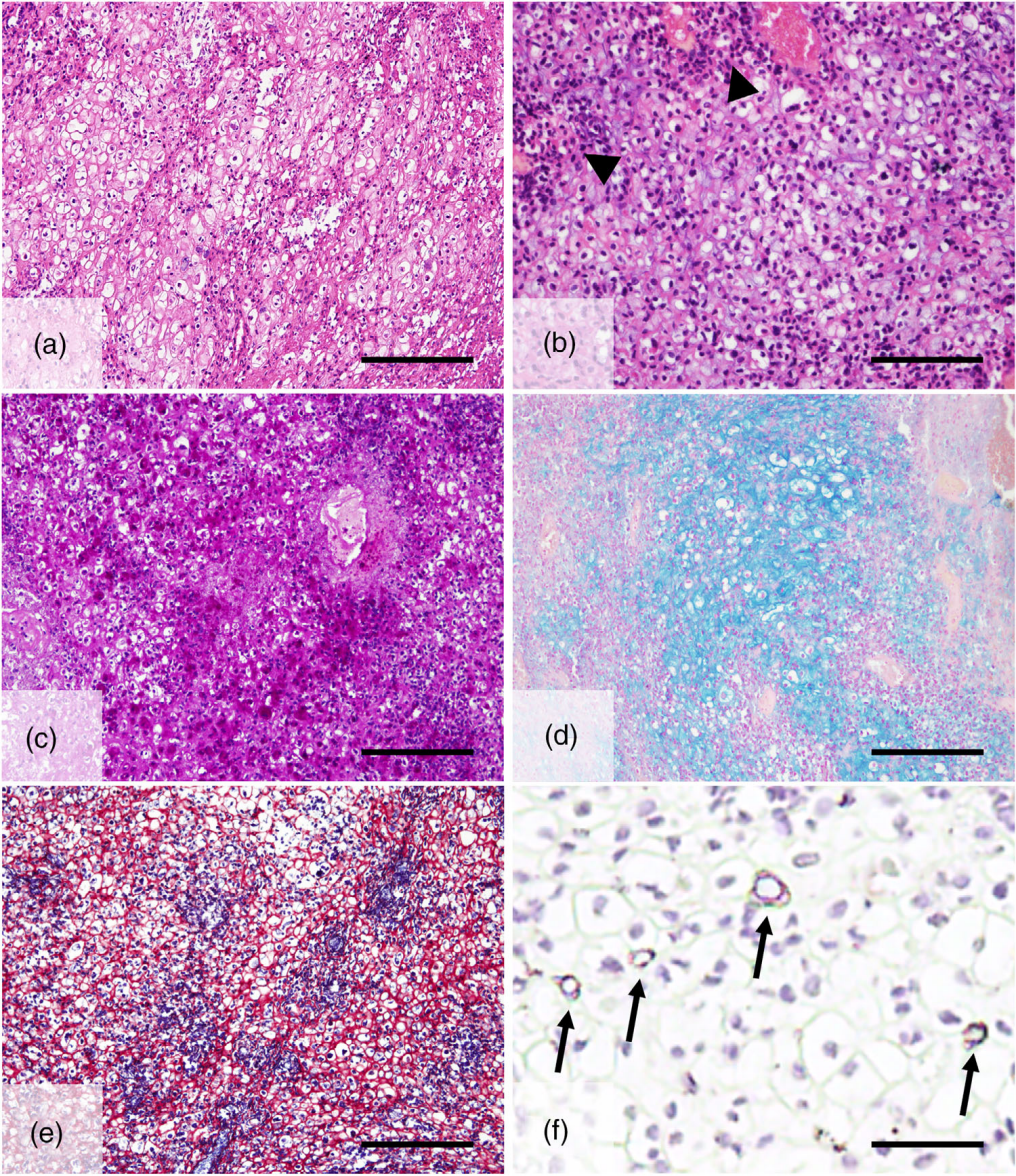
Microscopic examination of clear cell variant mucoepidermoid carcinoma, salivary gland, grey wolf (*Canis lupus*). (a) Low‐power view of the tumour. The tumour was composed predominantly of clear cells. H&E staining, scale bar is 100 μm. (b) High‐power view of the tumour. Clear cells had variably sized clear cytoplasm and hyperchromatic nuclei. Some of the tumour cells showed relatively small acidophilic cytoplasm regarded as intermediate cells (arrowhead). H&E staining, scale bar is 50 μm. (c) Tumour cells exhibited mucin in the cytoplasm. Much of the staining was positive for PAS and (d) alcian blue at a pH of 2.5, scale bar is 100 μm. (e) The cytoplasm of the tumour cells was negative on phosphotungstic acid haematoxylin staining. PTAH, scale bar is 100 μm. (f) Some tumour cells were positive for vimentin (black arrows). Immunohistochemistry, scale bar is 25 μm.

Normal salivary glands are composed of several types of cells, and tumours can arise from multiple cell types. Moreover, the histological characteristics of the salivary duct epithelium rapidly change in non‐neoplastic conditions such as inflammation as well as in tumours (Carberry et al., 1988). Because salivary tumour tissue has various cytological features, it is difficult to classify primary salivary gland tumours.

MEC is categorised into two basic types: ‘classical’ and ‘variant’. While classical MEC is composed of clearly recognisable mucous, epidermoid and intermediate cells in variable proportions, variant MEC shows the presence of ≥80% non‐classical cell types, including clear cell, eosinophilic or squamoid cells (Ettl et al., 2012; Schwarz et al., 2011). Generally, clear cells account for approximately 10% of the cell population of MEC (Ellis, 1998), and they have a characteristic clear cytoplasm due to cytoplasmic accumulation of non‐staining components such as glycogen, lipid, or mucins (Terada et al., 2004). The diagnosis of these intracellular components may be facilitated by special stains such as PAS, PAS‐D, alcian blue (pH 2.5), mucicarmine or oil red O. In rare instances, clear cells may constitute a large area of the tumour, thus giving rise to the clear cell variant of MEC. Clear cell tumours are recognised in several salivary gland tumours, including MEC, acinic cell carcinoma, clear cell oncocytoma, clear cell adenocarcinoma, epithelial‐myoepithelial carcinoma, myoepithelial carcinoma and metastatic renal cell carcinoma (Terada et al., 2004). All clear cell tumours of salivary glands, except clear cell oncocytoma, are malignant (Fletcher, 2007). Therefore, histopathological examination is an important aspect of the diagnosis of these clear cell tumours. H&E staining is the gold standard method used for diagnosing salivary gland tumours, and various staining methods improve the accuracy of such analysis (Nagao et al., 2012). In the present case, demonstration of intracellular mucins and undifferentiated intermediate cells on H&E, mucin staining (PAS, PAS‐D and alcian blue) and immunohistochemical staining helped to confirm the diagnosis of MEC with clear cell features.

Histologically MEC is divided into low, intermediate, and high grades, based on the microscopic morphology of the tumour cells (Shafique et al., 2020). Low‐grade, also called well differentiated, MEC appears similar to the normal salivary gland. Well‐developed cystic structures and mucus cells are prominently demonstrated in low‐grade MEC. Intermediate‐grade or moderately differentiated MEC has characteristics in between low‐ and high‐grade disease. Intermediate cells that form solid islands predominate, and intermediate grade tumours have fewer and smaller cysts than low‐grade tumours. High grade MEC is also called poorly differentiated MEC. Excessive proliferations of epidermoid and intermediate cells are found in high grade MEC. Furthermore, higher degrees of atypia, anaplasia, increased mitotic rate, necrosis, perineural and lymphovascular invasion are easily identified. Although the origin of the present tumours was unclear due to severe infiltration and extension into the surrounding tissue structures, based on the histopathological features, the wolf was diagnosed with high‐grade clear cell variant MEC, and we assumed that this tumour originated from the parotid gland. This type of tumour has been reported in some domestic species and laboratory animals (Hammer et al., 2001; Ishikawa et al., 1998; Nau et al., 2017). In wildlife, however, salivary gland tumours have rarely been reported. Only one case of high‐grade MEC in wildlife was previously described in the veterinary literature, in a male lion (*Panthera leo*) (Dorso et al., 2008).

In the lion case, microscopic features were similar to our present study. In addition, as with our results, MEC in lion showed positive for vimentin and negative for alpha‐SMA. However, in the case of the lion, CKs were positive. CKs are intermediate filaments that are used for the diagnosis and classification of epithelia (Moll et al., 1982). Previous studies used CKs to evaluate the patterns in MEC, and there is a published paper that analysed the immunoprofile of CKs in different cellular types of MEC (Azevedo et al., 2008). Azevedo et al. indicated that CK 6, 7 and 8 were mainly immunopositive in clear cell type of human MEC cases. Our CKs antibody is a cocktail of anti‐CK antibodies, AE1 and AE3(MNF‐116, Dako, CA, USA). It has broad spectrum of reactivity against various sized and types of CK. However, in our case, cocktail CKs did not work. As the antibody's reactivity is not confirmed in all species, we assume that our antibody did not work in wolf species. Therefore, it is difficult to conclude that the clear cell variant mucoepidermoid carcinoma in wolf is negative for CKs. In this regard, we suggest the application of CK 6, 7, and 8 antibodies that show strong reactivity in clear cell type rather than cocktail antibodies.

At five months after showing the first clinical sign, the wolf died. Metastasis to other organs and regional lymph nodes was not observed. The difficulty to eat or loss of appetite due to tumour and facial oedema may result in nutritional disorders (Solheim et al., 2014). Or in this case, as the tumour induced local osteolysis, it may be progressed to tumour‐related hypercalcemia (Goldner, 2016). Also decreased immunity and secreted cytokines due to tumours may be the cause of death (Gonzalez et al., 2018).

The veterinarian in charge suspected an infection, prescribed only antibiotics and analgesics and observed the prognosis. Until the wolf died, the veterinarian did not suspect a tumour, and as the facial swelling was progressed, the veterinarian regarded the condition as an abscess arising from local infection. According to the Wildlife Protection And Management Act, Act On The Management Of Zoos And Aquariums, Animal Protection Act in the Republic of Korea, the veterinarian did not neglect animals suffering a disease. According to the results of the pathological examinations, it was confirmed that it was a highly malignant tumour. Furthermore, zoo veterinarian could not make the accurate diagnosis until the death of the animal. A good prognosis could not be expected because the wolf was an old (14‐year‐old) along with a malignant tumour, but if the veterinarians suspected the tumour at early stage, they would have focused on reducing the pain. Animals kept in zoos have a longer average life expectancy compared to those in wild conditions because they do not struggle for survival and are constantly monitored and treated (Müller et al., 2010). In addition, the incidence and diagnosis rate of tumours are increasing due to prolonged lifespan and frequent medical monitoring (Hubbard et al., 1983). Therefore, this case represents a great opportunity to learn more on tumours in zoo and captive wildlife, and to promote animal welfare through practicing timely and accurate diagnosis with appropriate treatment plans.

Besides, data on tumours in wolf are limited, and until now, a squamous cell carcinoma of the tonsil and pulmonary neuroendocrine tumour were reported (Shiraki et al., 2017; Teifke et al., 2005). In this regard, our present case represents a valuable addition to the knowledge on neoplasia in wolf.

In summary, we reported an extremely rare case of high‐grade clear cell variant MEC in a wolf. This is the first report in a wolf in the veterinary literature.

## AUTHOR CONTRIBUTIONS

Bumseok Kim: Conceptualisation; data curation; formal analysis; funding acquisition; investigation; project administration; supervision; writing – review & editing. Myeon‐Sik Yang and Hyejin Yun: Data curation; formal analysis; investigation; methodology; visualisation; writing – original draft; writing – review & editing. Ki Chang Lee: Data curation; formal analysis. Chae Woong Lim: Supervision; writing – review & editing.

## CONFLICT OF INTEREST

The authors declare no conflict of interest with respect to the publication of this manuscript.

### ETHIC STATEMENT

This case and procedures were reviewed and approved by the Animal Ethics Committee of Jeonbuk National University.

### PEER REVIEW

The peer review history for this article is available at https://publons.com/publon/10.1002/vms3.909.

## Data Availability

The data that support the findings of this study are available from the corresponding author upon reasonable request.
